# Dynamic Metabolic Footprinting Reveals the Key Components of Metabolic Network in Yeast *Saccharomyces cerevisiae*

**DOI:** 10.1155/2014/894296

**Published:** 2014-01-29

**Authors:** Pramote Chumnanpuen, Michael Adsetts Edberg Hansen, Jørn Smedsgaard, Jens Nielsen

**Affiliations:** ^1^Department of Zoology, Faculty of Science, Kasetsart University, Bangkok 10900, Thailand; ^2^Institut Pasteur Korea, Sampyeong-dong 696, Bundang-gu, Seongnam-si, 463-400 Gyeonggi-do, Republic of Korea; ^3^National Food Institute, Technical University of Denmark, Mørkhøj Bygade 19, 2860 Søborg, Denmark; ^4^Department of Chemical and Biological Engineering, Chalmers University of Technology, Kemivägen 10, 412 96 Gothenburg, Sweden

## Abstract

Metabolic footprinting offers a relatively easy approach to exploit the potentials of metabolomics for phenotypic characterization of microbial cells. To capture the highly dynamic nature of metabolites, we propose the use of dynamic metabolic footprinting instead of the traditional method which relies on analysis at a single time point. Using direct infusion-mass spectrometry (DI-MS), we could observe the dynamic metabolic footprinting in yeast *S. cerevisiae *BY4709 (wild type) cultured on 3 different C-sources (glucose, glycerol, and ethanol) and sampled along 10 time points with 5 biological replicates. In order to analyze the dynamic mass spectrometry data, we developed the novel analysis methods that allow us to perform correlation analysis to identify metabolites that significantly correlate over time during growth on the different carbon sources. Both positive and negative electrospray ionization (ESI) modes were performed to obtain the complete information about the metabolite content. Using sparse principal component analysis (Sparse PCA), we further identified those pairs of metabolites that significantly contribute to the separation. From the list of significant metabolite pairs, we reconstructed an interaction map that provides information of how different metabolic pathways have correlated patterns during growth on the different carbon sources.

## 1. Introduction

The word metabolism comes from Greek *metabolé* which means change or transformation, and in fact the levels of most cellular metabolites change with half times of minutes, seconds, or even faster. During the past years the complexity and dynamics nature of the metabolites have become increasingly apparent [1–6]. The comprehensive analysis of a large set of metabolites, now referred to as metabolomics, present in a biological sample, has emerged as an important tool in functional genomics and systems biology [7, 8]. Since it is “downstream” of central dogma (not like transcriptome and proteome at higher cascade), the metabolome should show greater effects of genetic or physiological changes [9, 10] and is therefore much closer related to the phenotype expressed by an organism [11]. Metabolic fingerprinting (an analysis of intracellular metabolites profiling) and metabolic footprinting (an analysis of extracellular metabolites profiling) have succeeded in experimental characterization of genetic mutants on the basis of combined intracellular and extracellular metabolite data measured by mass spectrometry (MS) [5, 8, 11, 12]. However, the measurement of extracellular metabolites, often referred to as footprinting [4, 11], represents several advantages over the analysis of intracellular compounds, often referred to as metabolic fingerprinting [13], for microbial cultures. Although the functional analysis using metabolic fingerprinting can classify the different phenotypes of several mutants, it is difficult to scale up for high-throughput screening of many mutants as it is quite time-consuming and subject to technical difficulties caused by the rapid turnover and the need of quenching and extracting of metabolites from the extracellular space [5, 11].

Analysis of metabolite profiles can be done using different analytical techniques, but recent technical advances in MS have brought this technology to the forefront among methods for metabolome analysis due to the high sensitivity and separation efficiency [13, 14]. Gas chromatography coupled to MS (GC-MS) is the most extensively used technique in metabolome analysis [15], as it is effective for resolving complex biological mixtures and hereby enable reliably identification of the analyzed compounds. However, GC-MS can only analyze metabolites which are stable at the high temperatures present in GC and they have to be volatile or can be made volatile upon chemical derivatization. Most metabolites are not volatile and many of them are not stable at high temperature or cannot be derivatized. Recently, Direct Infusion-Mass Spectrometry (DI-MS) has been reported as an alternative to GC-MS and it seems to be an ideal analytical tool for high-throughput metabolome analysis [16–20]. The most significant feature of ESI mode for DI-MS is that it is a very soft ionization technique which, in many cases, will produce protonated molecular species (in positive ESI) for a broad range of different compounds with very high sensitivity [19]. With high sensitivity and atmospheric pressure ionization, DI-MS can analyze the majority of metabolites in the sample in a few seconds without any chromatographic separation [16, 17, 19, 20]. An obvious extension of a DI-MS approach is to store the spectra in a database using the database software included with most instruments. This will give a sample identification database rather than compound identification as anticipated by the manufactures [15, 17, 19, 21, 22].

Here we present the use of dynamic metabolic footprinting analyzed by DI-MS for phenotypic information of yeast. Using novel data analysis methods we show that it is possible to extract the key information about the metabolism from footprinting data when yeast is grown on different carbon sources. The data analysis is based on identification of significantly correlated metabolites over time, which corresponds to flux-ratios for different metabolites. The advantage of this approach is that it is not sensitive to a single time point and, therefore, better allows for analysis of mutants having different growth rates.

## 2. Materials and Methods

### 2.1. Reagents

All reagents used for metabolite analysis and the medium for yeast cultures were prepared using analytical grade ingredients. Methanol, acetonitrile, and formic acid used for mass spectrometry were obtained from Sigma-Aldrich (St. Louis, MO, USA). MilliQ-purified water was used during the sample preparation for HPLC and DI-MS analyses.

### 2.2. Yeast Strain

The *S. cerevisiae* strain BY4709 (MAT *αura3*Δ0) obtained from the European* S. cerevisiae* archive for functional analysis (EUROSCARF, Frankfurt, Germany) was used in this study.

### 2.3. Medium

The strain was grown in a minimal synthetic medium supplemented with a metabolite cocktail according to Allen et al. [11] (see Table 1). The metabolite cocktail contained a selection of amino acids, organic acids, and organic bases. The medium was based on yeast nitrogen base (YNB) without amino acids (BD Difco, Franklin Lakes, NJ, USA) and the metabolite cocktail was composed of L-arginine, L-aspartate, L-glutamate, L-histidine, L-leucine, L-lysine, L-methionine, L-serine, L-threonine, L-tryptophan, L-valine, citrate, fumarate, malate, pyruvate, succinate, cytosine, and uracil. All compounds from the metabolite cocktail had a concentration of 1 mM in the final medium. Cultivations were performed with 3 different carbon sources, that is, glucose, glycerol, and ethanol. Each carbon source is dissolved in water, autoclaved separately, and finally added to give a final concentration 0.67 C-mole/L corresponding to (20.00 g/L glucose, 15.34 g/L ethanol, or 20.45 g/L glycerol).

### 2.4. Cultivation Conditions

Cultivations were performed in 5 replicates in 500 mL baffled shake flasks (150 rpm), at 30°C. Each flask contained 100 mL of footprinting media prepared according to Allen et al. [11] and closed with cotton plugs. During cultivation, the culture purity was monitored on a regular basis by phase contrast microscopy and they east growth was monitored by OD measurements at 600 nm.

### 2.5. Cell Mass Determination

Biomass dry weight was determined by filtering a known volume of fermentation broth, approximately 5 mL, through a dried, preweighed nitrocellulose filter (Sartorius Stedim Biotech S.A.) with a pore size 0.45 *μ*m. The residue was washed twice with distilled water. The filter was dried to constant weight in a microwave oven at 150 W for 10 min, cooled in a desiccator, and the weight was measured. Besides, the optical density was determined at 600 nm using a Genesis 20 (Thermo spectronic), spectrophotometer. Samples were diluted with water to obtain OD measurements in the linear range of 0.1–0.5 OD units.

### 2.6. Analysis of Culture Media by HPLC

Samples harvested from the cultivation broth were immediately filtered through a 0.45 *μ*m pore-size cellulose acetate filter (VWR) and stored at –20°C until analysis. Glucose, glycerol, ethanol, succinate, and acetate concentrations were determined by HPLC analysis using an Aminex HPX-87H column (Biorad, Hercules, CA) and all the conditions were set following Zaldivar et al. [23]. Briefly, the separation was performed at 45°C, with sulfuric acid (5 mM) at a flow rate of 0.5 mL/min as the mobile phase. Subsequent determinations of yield coefficients for extracellular metabolites as well as biomass were based on linear regressions of their concentration as a function of the residual glucose concentration in the exponential growth phase.

### 2.7. Sampling Procedure

The yeast strain BY4709 was grown on three different carbon sources: glucose, ethanol, and glycerol. For each carbon source 5 replicates were carried out and the samples were taken every 3 hours from 3 hr until 30 hr such that they represented different growth phases for growth on the different carbon sources. Two-milliliter samples were centrifuged (8,000 g, 10 min) and the supernatant was stored at –18°C until further DI-MS analysis.

The supernatants were diluted fivefold with acetonitrile right before they were analyzed by DI-MS.

### 2.8. Direct Infusion-Mass Spectrometry Analysis

**Figure 1:**
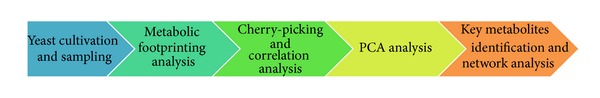
The pipeline for our dynamic metabolic footprinting process. Different perturbations are imposed on the microorganism, for example, growth on different carbon sources or parallel analysis of different mutants, and the growth profile is recorded using at least 10 samples. The samples are processed and analyzed using DI-MS. The resulting spectra are processed to obtain correlation between different metabolites analyzed.

The sample preparation for the dynamic metabolic footprinting analysis was performed as illustrated in the metabolic footprinting pipeline in Figure 1. The supernatants were diluted fivefold with acetonitrile right before the injection. The DI-MS analysis was performed on a system setup with an Agilent 1100 microflow HLPC pump, LC-Packings autosampler coupled to a Micromass (Waters, Manchester) Q-tof system with an electrospray ionization interface. The instrument was tuned for maximal sensitivity at low flow rate and minimal fragmentation using leucine-enkephalin, followed by external calibration using a mixture of PEG200 and 400 in acetonitrile-water. The samples were diluted fivefold in acetonitrile (with 1 *μ*g/*μ*L leucine-enkephalin as an internal standard mass reference) and centrifuged at 10,000 g for 1 min. The supernatants were transferred into 200 *μ*L HPLC vial inserts and the vials were placed in the autosampler. The sequence of samples was randomly injected in order to decrease the effects from instrumental bias. The samples were analyzed by infusion of 5 *μ*L sample into the ESI source of Q-tof MS at a flow rate of 20 *μ*L/min. A carrier flow of methanol was used at a rate 15 *μ*L/min from the LC-pump through the autosampler; just before the ion source a flow of 2% formic acid in water was fed into the solvent stream from a syringe pump at a rate of 5 *μ*L/min using a t-piece giving a combined flow of 20 *μ*L/min of 75% methanol-water with 0.5% formic acid going into the source. Mass spectra were acquired in both positive and negative mode and data were collected for 3 min/sample between 50 and 1000 Da/e at a rate of one continuum scan/second. The Q-tof conditions were the following: capillary voltage 3,000 V in positive mode and 2,600 V in negative mode, desolvation temperature 150°C, dry gas, desolvation gas at 300 L/hr, nebulizer flow 20 L/hr, source temperature 90°C, and cone voltage optimized to minimal fragmentation approximately 40 V in positive mode and 30 V in negative mode.

### 2.9. Data Analysis

Initially, the raw data were processed according to the method described by Hansen and Smedsgaard [24]. The MS data (as shown in Figure S1A) were preprocessed in the following way: for each sample, the elution profile were detected, 40 continuum scans (1 s/scan) were summarized to a single continuum spectrum to reduce noise, followed by background subtraction and calculating the centroid spectrum (Figure S1B). Then, all data were normalized based on the ion count of leucine-enkephalin (*m/z* 556.2771) internal standard to reduce technical variability from the instrumental bias [24]. The result was a matrix where each row corresponded to a sample and each of the columns to a metabolite. The width of each column roughly corresponded to 0.5 *m/z*.

In the following, two criteria were applied for selecting (*cherry-picking*) the metabolites.

1. The first criteria dealt with screening for the single metabolites in each injected sample showing proper changes over time (either decreasing or increasing or a combination of both). Since the changing pattern can either be linear or nonlinear (e.g., exponential or sigmoidal pattern), the data smoothness step was required for the nonlinear function by fitting to the (standardized) time profiles. In the later step, those selected metabolites which have the actual trend in the profiles (noise excluded) will further be analyzed.
2. For the next criteria, we identified metabolite pairs which show the covariance across time. Each metabolite was paired up and plotted against each other to investigate the correlation pattern of individual pair and the regression line was fitted to the data. Finally, the correlation (*R* ^2^ of the regression line) and the *P* value were calculated.

The intercept, slope (which is equivalent to what is often referred to as yield coefficients), *R* ^2^, and the *P* value of the regression line for each metabolite were stored in a matrix of dimension as the number of metabolites.

Since many of the identified metabolites are related and covary we used only the selected metabolites to analyze the variance present in data. Whereas ordinary Principal Component Analysis (PCA) [25] is widely used in data processing and dimensionality reduction, PCA in general suffer from the fact that each component is a linear combination of all the original variables. Even if some variables contain random noise, those will be assigned weights (loadings) in the linear combination. Thus, it is often difficult to interpret the results. As for ordinary PCA, Sparse PCA [26] finds sets of Sparse vectors (having a relatively small number of nonzero elements) for use as weights (loadings) in the linear combinations while still explaining most of the variance present in the data. For all samples, based on the *P* value calculated for each pair of ions, we could generate a symmetric matrix of correlations with the ions along both axes. The upper triangular part of the matrix was extracted and unwrapped (Figure S2). Based on the matrix WT (1) × ESI-mode (2) × Source (3) × Rep (5) rows and (*N* − 1)*N*/2 columns, where *N* is the number of ions present, we investigated the variation (informational) content by Sparse Principal Component Analysis and further identified metabolites that additionally correlated in response to time for growth on the different carbon sources.

## 3. Results and Discussions

Metabolic footprinting is traditionally based on analysis at a single time point. Therefore, the analysis becomes very dependent on the growth rate of the organism and also on factors such as inoculum size. In order to circumvent this problem we developed a pipeline for dynamic metabolic footprinting (Figure 1). This involved both HPLC and MS analysis of extracellular samples during the fermentation.

### 3.1. Cultivation Profile

Based on HPLC measurements of the carbon sources and metabolic products the overall kinetics of substrate uptake could be determined. For the glucose cultures, the cells took approximately 12 hours to finish their exponential phase with a glucose consumption rate of 0.304 g glucose/gDW/h (see Table 2 and Figure 3). In contrast, the cells growing on a nonfermentative carbon source took 21 hours to finish their exponential phase with a consumption rate of 0.027 g ethanol/gDW h and 0.048 g glycerol/gDW/h, respectively. Even though they were grown in different initial concentrations in terms of g substrate/L the C-mole amounts were exactly the same (0.67 C-mole/L) for all three carbon sources. Comparing the specific consumption rate in terms of C-mole (see Figure 3 and also Table 2), the cells could consume glucose approximately 10 times faster than ethanol and glycerol (i.e., 10.133, 1.117, and 1.564 C-mmole of carbon/gDW h for glucose, ethanol, and glycerol, resp.). Not surprisingly, yeast cells preferred glucose which is a fermentable carbon source more than the 2 nonfermentable carbon sources.

### 3.2. Dynamic Metabolic Footprinting and DI-MS Analysis

There is as mentioned in the introduction an interest in using metabolic footprinting as the turnover rate for extracellular metabolites is much longer, than that for intra-cellular metabolites (fingerprinting) and footprinting does not require any complicated procedure for the quenching and extraction steps. However, using a single data point can cause problems with capturing the phenotype of different mutants, and the approach proposed here may therefore allow for wider use of metabolic footprinting. Here we demonstrate high-throughput metabolic footprinting using DI-MS, which allow us to cover more than 2,000 ion peaks (both from positive and negative ESI mode), which cover many metabolites in different pathways to be analyzed at the same time. From the DI-MS analysis results and its integrated signal spectra (Figures S1A and B), we pointed out how the technique was so consistent giving such a high reproducibility in all 5 biological replicates. Even though it is complicated to interpret biological meaning from thousands of ion peaks, we here demonstrate the cherry-picking criteria to identify a list of single metabolites that showed significant change over time. Thus, our method is an unbiased strategy to get to the key metabolites.

The DI-MS analysis showed that amino acids were best detected in positive mode, probably due to the amino group. The typical base peak ion was [M + H]^+^ and [M + CH_3_CN + H]^+^.

### 3.3. Key Metabolites (Cherries) Picked up from MS Spectra

After conversion and alignment of the data according to Hansen and Smedsgaard [24], each of the metabolites was first analyzed for trends over time. The resulting matrix had 300 rows (one for each sample) and approximately 1500 columns. After binning the data, as part of the initial procedure, columns containing no data were removed.

**Figure 2:**
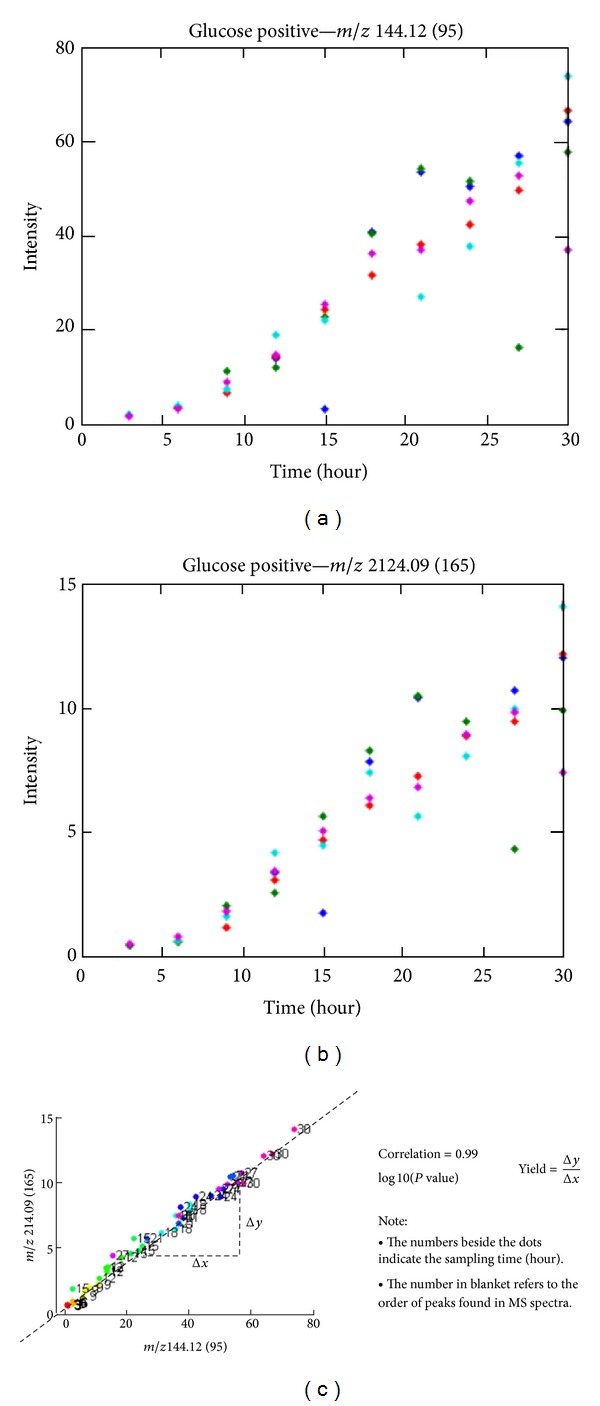
The cherry-picking process illustrated. Two upper figures show two selected metabolites during the first step, and the lower figure shows their corresponding correlation in time. Both metabolites will be picked. Slopes (yields) in the matrix that had a *P* value less than 0.01 were set to zero.

The first cherry-picking criteria identified a list of single metabolites that showed the proper change over time. After this step, the number of columns in the matrix was reduced to 145 metabolites. Two examples of identified metabolites can be seen in the top two figures in Figure 2.

As the next criteria, the degree of covariance over time was calculated. Figure 2 (bottom picture) illustrates the process, where the two metabolites were picked during the first selection step, and shows covariance across time. For all metabolite pairs, the intercept, slope (yields), *R* ^2^, and the *P* value of the regression line for each metabolite were stored in a matrix of dimension as the number of metabolites.

### 3.4. Standard and Sparse PCA Analysis

In order to evaluate the information present in the matrix of metabolite pairs, we initially analyzed the variation in yields by PCA to identify the key metabolites which are significantly different between the sample groups and to how well they can separate growth on the different carbon source from each other.

The upper triangular part of the matrix of the yields was extracted and unwrapped. Based on the matrix columns as shown with the cherry-picking method (Figure 2), we calculated the PCA loading for PCA analysis.  Figure 4 illustrates the scores and loadings for the first two principal components using ordinary PCA. As can be seen from the plots, the yields of the selected metabolite pairs show a nice separation of the carbon sources. Still, when looking at the loadings we see that all metabolite pairs are assigned weights.

Sparse PCA was used to obtain sets of Sparse vectors for weights (loadings) in the linear combinations while explaining most of the variance present in the data.  Figure 5 illustrates the effect of using of Sparse PCA rather than ordinary PCA on the loadings. We see that most of the metabolite pairs have been assigned zero weight, whereas only a few pairs have been assigned a weight. Although most of the weights in the loadings are zero, we still see the same grouping into carbon sources as when using ordinary PCA.

As can be seen from the Sparse PCA, a few pairs of metabolites can separate the data into distinct carbon sources. Next, we wish to investigate those metabolites in further detail. From the reconstruction of the correlation networks it was found that pairs from PC1 are most important as they generate the largest network. PC1 segregates the signal from glucose from the other two carbon sources (ethanol and glycerol). The size of key metabolites network represents the level of differences in dynamic pattern among the sample groups [6, 21].

### 3.5. Identification of the High Correlated Ion Pairs

Pairs of MS ions that correlated over time were identified as those the Sparse PCA were assigned weights. Only the metabolite pairs from the loadings corresponding to the Sparse PC1 and Sparse PC2 were extracted. There were a total of 48 pairs, which corresponds to correlation between 44 ions as key metabolites: 24 pairs between 17 metabolites for PC1 and 3 pairs between 3 metabolites for PC2 in positive ESI mode (see Table 3), and 17 pairs between 19 metabolites for PC1 and 4 pairs between 5 metabolites for PC2 in negative ESI mode (see Table 4). A few metabolites were identified both in positive and negative ESI mode, and the number of metabolites identified was 45. For each ion the corresponding metabolite was identified using the database developed by Højer-Pedersen [17], and a list of the identified metabolites for the two modes are given in Tables 5 and 6. Moreover, the fragmentation patterns from ESI-MS of each metabolite were also carefully considered and compared according to the Golm metabolome databases [15] to confirm the possible calculated mass per charge. For most of the ions there is a unique identification of the corresponding metabolite, but for some ions more than one metabolite has the corresponding mass, for example, for the ion 179.0353 *m/z* there are three potential candidates for the corresponding metabolite, namely, 3-(4-hydroxyphenyl) pyruvate, glucose, and myo-inositol. In this particular case glucose is the most likely metabolite and it was chosen, but cases where there could not be made a clear assignment both metabolite names were used.

PC1 ions primarily separate the glucose samples from the ethanol and glycerol samples and PC2 mainly separates the ethanol samples from the glycerol samples. The metabolite pairs from PC1 represent a network as illustrated in Figure 6, and the metabolite pairs from PC2 represent two networks as illustrated in Figure 7. In these networks the edges represent correlations between the corresponding key metabolites. It is observed that most of the key metabolites are amino acids that are linked to cyclic AMP or cyclic dAMP, but glucose is also clearly correlated to several metabolites. The color code indicates the slope with green indicating decreasing concentration with time and red increasing concentration with time. A few metabolites do not change much in concentration profile or do not have a monotonous concentration profile during the whole fermentation, for example, their concentration are increasing in one part of the fermentation and decreasing in another part of the fermentation, and these are marked blue in the networks. It is interesting to note that there is a positive correlation between cAMP and most amino acids, whereas the glucose concentration is negatively correlated with the concentration of several metabolites, for example, glycerol.

**Figure 3:**
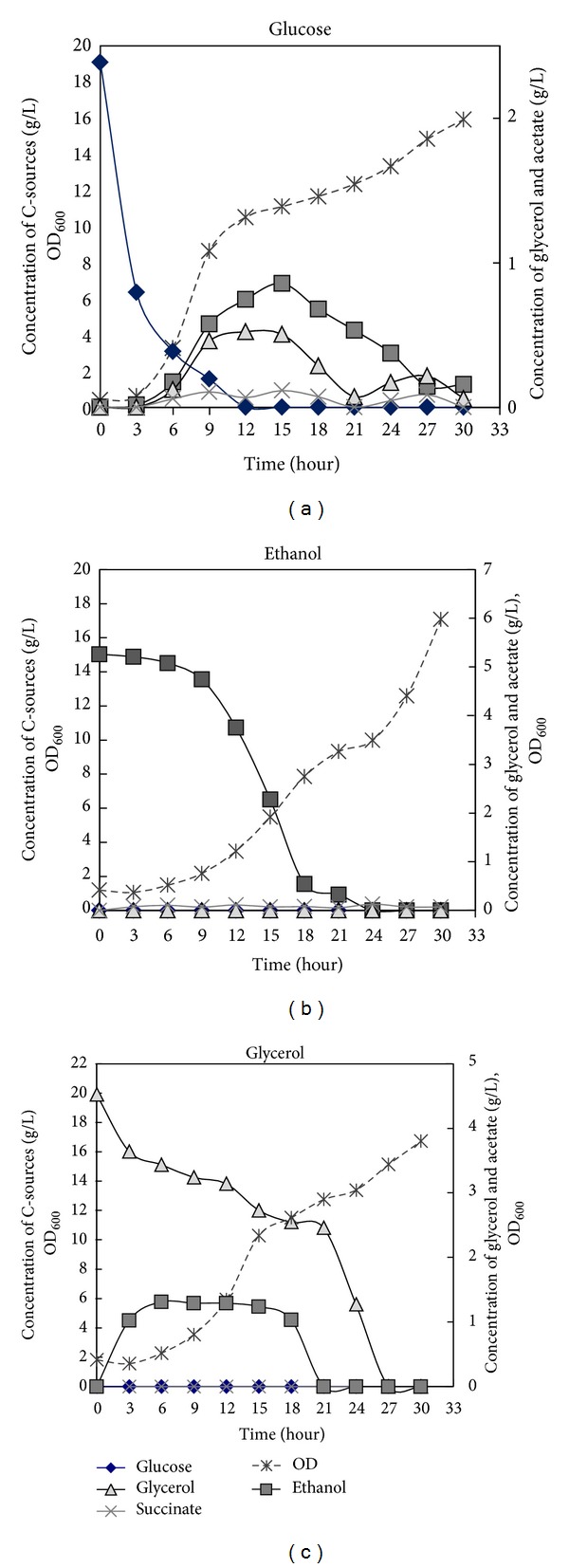
Profiles of growth (OD_600_) and metabolite concentrations; glucose, glycerol, succinate, and ethanol of yeast BY4709 during the fermentationmeasured by HPLC. The plots show fermentation profile grown in different carbon sources; glucose (a), ethanol (b), and glycerol (c).

**Figure 4:**
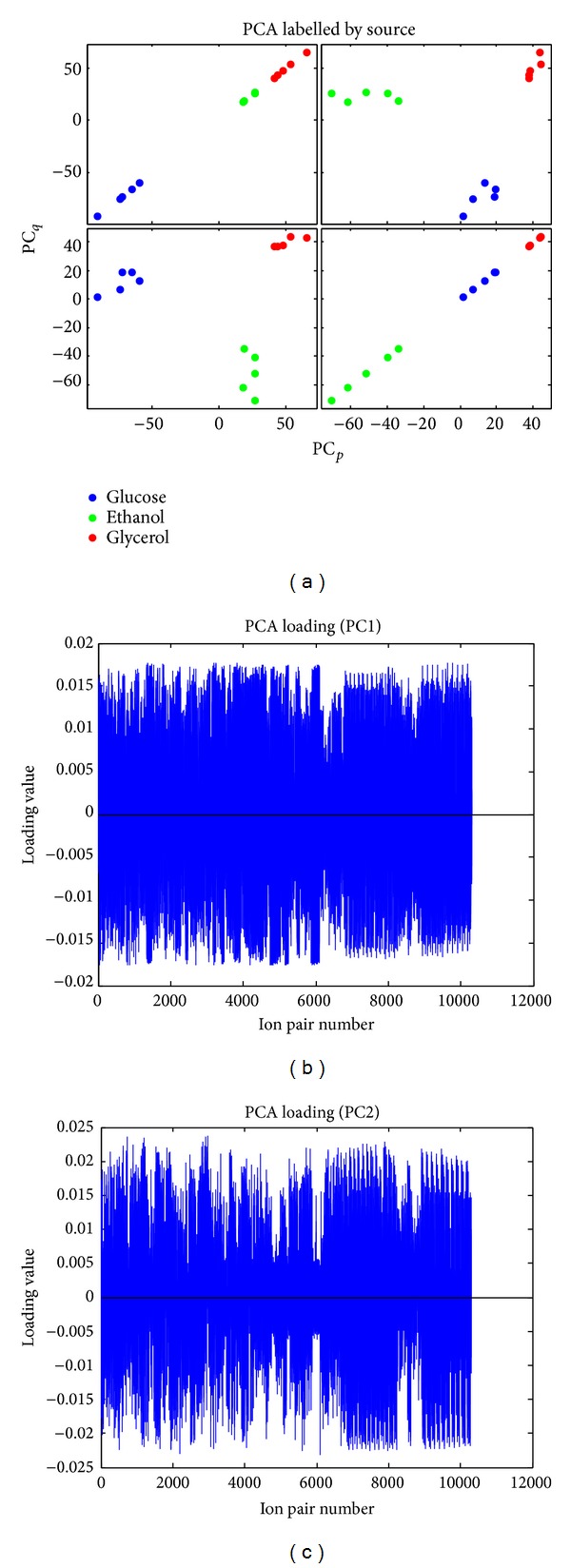
Standard PCA and loading show the clustering of different C-source cultures (including both ESI modes) and 5 replicates based on yield/slope of metabolite profile.

**Figure 5:**
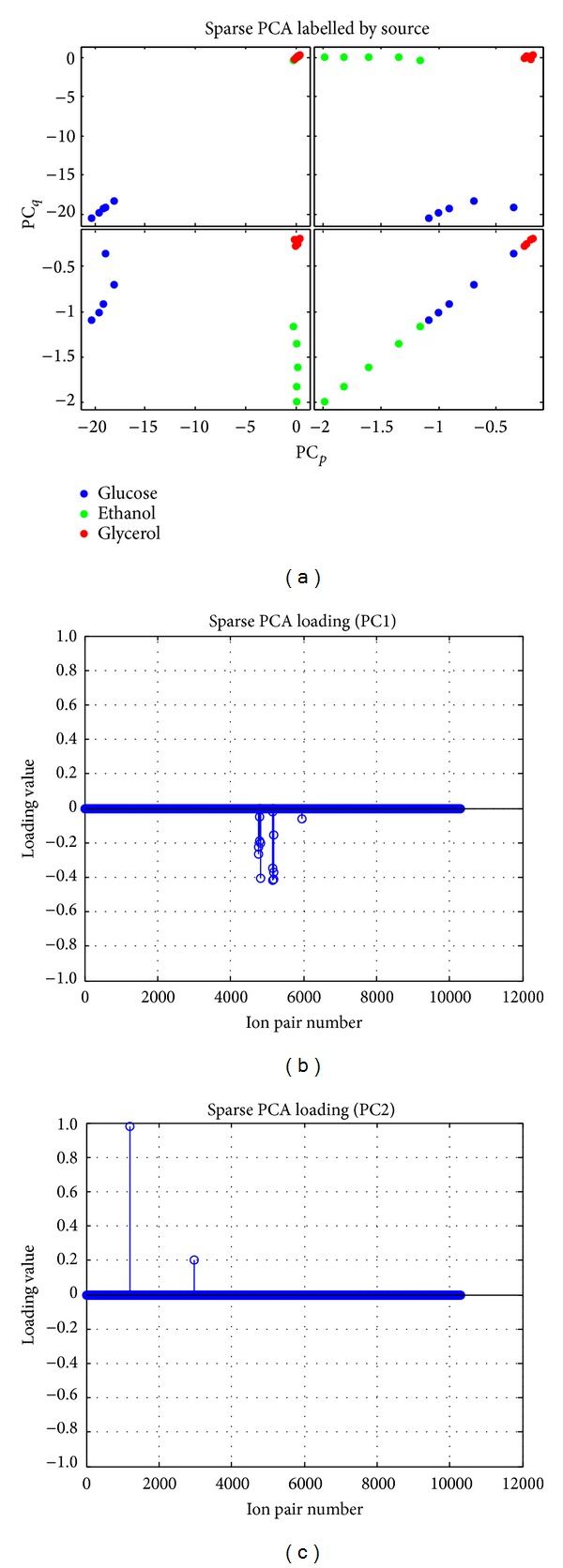
Sparse PCA and loading show the clustering of different C-source (including both ESI modes) and 5 replicates based on yield/slope of metabolite profile.

The PCA results, both ordinary PCA, and Sparse PCA showed that the main variance in the data is caused by the 3 different C-sources: glucose, ethanol, and glycerol. The Sparse PCA finds sets of Sparse vectors for use as weights in the linear combinations while still explaining most of the variance present in the data. It is built on the fact that PCA can be written as a regression-type optimization problem, with a quadratic penalty; the lasso penalty (via the elastic net) can then be directly integrated into the regression criterion, leading to a modified PCA with Sparse loadings [26]. According to the results we obtained from both ordinary PCA (Figure 3) and Sparse PCA (Figure 4), it is clearly shown that the Sparse PCA gives a better clustering among the groups compared to the normal PCA results. Focusing on the consistency of this analysis, the results from PCA indicated that the main variance in the data is caused by the different C-sources with high reproducibility. The 5 replicates are separated into the same cluster based on the dynamic metabolic profile which is promising.

### 3.6. Key Components of Metabolic Network of Yeast Grown on Different C-Sources

The key metabolites network on PC1 which contain more metabolite nodes compared to that on PC2 show the higher difference between glucose and ethanol or glucose and glycerol cultures while there are less differences between glycerol and ethanol cultures. In other words, the dynamic pattern of the glucose culture is more unique than the rest. So, we can obviously see that there are largest dynamic changes in metabolite concentrations during growth on glucose, which can be explained by the fact that glucose is a fermentative carbon source whereas there is purely respiratory growth on ethanol and glycerol.

**Figure 6:**
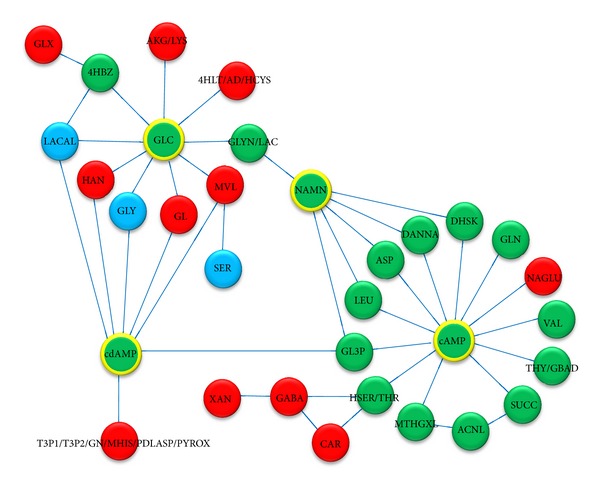
Key metabolite network on PC1; there are 32 key metabolites linking to each other with 4 hubs, that is, glucose, cyclic AMP, cyclic dAMP, and NAMN in PC1 network. These key metabolites can be used as PC1 to separate the sample taken from glucose culture from ethanol and glycerol cultures. Since there are 3 different patterns of changing in metabolite profile, these nodes are shown in 3 different colors; red (increasing), (decreasing), and blue (nonmonotonous or constant).

The 4 main hubs of the network are glucose, cyclic AMP, cyclic dAMP, and nicotinate-D-ribonucleotide (NAMN) as illustrated in the key metabolites network on PC1 (Figure 6).

Cyclic AMP and cyclic dAMP have been shown to regulate many different nutrient responses. The levels of these metabolites are linked to the biosynthesis of many amino acids and also affected by the concentrations of glucose and also amino acids [27]. Since fermentable sugars are specific stimulators for cAMP synthesis in yeast cells [28], the Ras/cAMP pathway is activated by both growth signal (e.g., glucose) and stress signals (e.g., UV radiation and starvation) [29]. Our finding from the correlation analyses is consistent with this as it shows that glucose and cAMP have the same dynamic pattern at all conditions.

**Figure 7:**
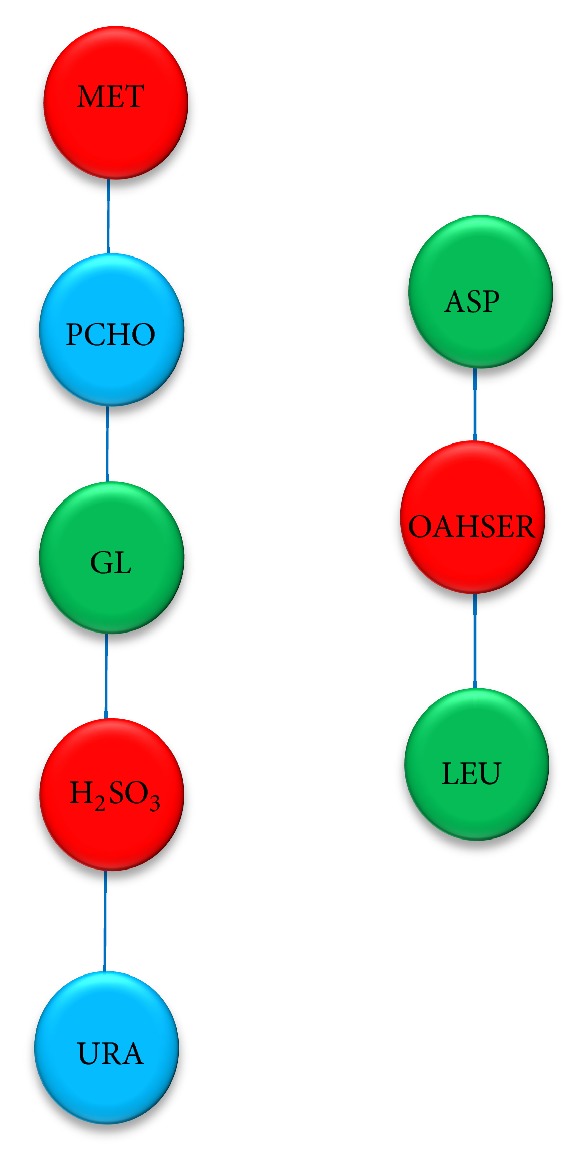
Key metabolites network on PC2; there are 8 key metabolites in PC2 network, 5 and 3 nodes in each subnetwork. These key metabolites can be used as PC2 to separate the sample group taken from ethanol culture from glycerol culture. Since there are 3 different patterns of changing in metabolite profile, these nodes are shown in 3 different colors; red (increasing), (decreasing), and blue (nonmonotonous or constant).

Since low cAMP concentration stimulates the uptake of and L-leucine [30], we can see that the uptake rate of leucine was increasing (the extracellular leucine level was decreasing), while the extracellular cAMP level was decreasing. From the key metabolites network on PC2 (Figure 7), we also found the link between methionine, glycine, 4-phospho-hydroxy-L-threonine, and pyridoxine. As the methionine is required for synthesis of phosphatidylcholine (PC) via methylation of phosphatidylethanolamine (PE) [31], these two metabolites have high correlation (sharing the same pattern) in correlation analysis result. In a similar case, 4-phospho-hydroxy-L-threonine (4HLT, the precursor to pyridoxal 5′-phosphate biosynthesis) was also linked to glycine, serine, and threonine metabolism and also pyridoxal phosphate synthesis pathway via cyclic AMP and cyclic dAMP.

In conclusion we here show that through dynamic metabolic footprinting it is possible to identify correlations between metabolites that can be used to provide new insight into possible regulatory structures. Furthermore, our correlation analysis may be used for identification of key biomarkers for specific phenotypes during dynamic growth conditions.

## Figures and Tables

**Table 1:** Media composition used for metabolic footprinting experiment.

	Group	Formula	Common name	MW	g/L
Yeast nitrogen base	Nitrogen source	(NH_4_)_2_SO_4_	Ammonium sulphate	132.13952	5.0198
	Vitamins	C_10_H_16_N_2_O_3_S	Biotin	244.31064	0.00002
		C_18_H_32_CaN_2_O_10_	Calcium pantothenate	476.53208	0.002
		C_19_H_19_N_7_O_6_	Folic acid	441.39746	0.002
		C_6_H_12_O_6_	Inositol	180.15588	0.01
		C_6_H_5_NO_2_	Niacin	123.1094	0.0004
		C_7_H_7_NO_2_	*p*-Aminobenzoic acid	137.13598	0.0002
		C_8_H_11_NO_3_·HCl	Pyridoxine hydrochloride	205.63878	0.00056
		C_17_H_20_N_4_O_6_	Riboflavin	376.3639	0.0002
		C_12_H_17_ClN_4_OS·HCl	Thiamin hydrochloride	337.26852	0.0004
	Trace elements	H_3_BO_3_	Boric acid	61.83302	0.05
		CuSO_4_	Copper sulphate	159.6086	0.00004
		KI	Potassium iodide	166.00277	0.0001
		FeCl_3_	Ferric chloride	162.204	0.0002
		MnSO_4_	Manganese sulphate	151.000645	0.0004
		Na_2_MoO_4_	Sodium molybdate	205.9171386	0.0002
		ZnSO_4_	Zinc sulphate	161.4716	0.0004
	Salts	KH_2_PO_4_	Potassium phosphate monobasic	136.085542	0.85
		K_2_HPO_4_	Potassium phosphate dibasic	174.175902	0.15
		MgSO_4_	Magnesium sulphate	120.3676	0.5
		NaCl	Sodium chloride	58.44276928	0.01
		CaCl_2_	Calcium chloride	110.984	0.1

Metabolite cocktail	Amino acids (all L-form)	C_6_H_14_N_4_O_2_	Arginine	174.20096	0.1742
		C_4_H_6_NO_4_K	Aspartate (monopotassium salt)	171.19304	0.1712
		C_5_H_8_NNaO_4_	Glutamate (monosodium salt)	169.1110893	0.1691
		C_6_H_9_N_3_O_2_	Histidine	155.15456	0.1552
		C_6_H_13_NO_2_	Leucine	131.17292	0.1312
		C_6_H_14_N_2_O_2_	Lysine (hydrochloride)	146.18756	0.1462
		C_5_H_11_NO_2_S	Methionine	149.21134	0.1492
		C_3_H_7_NO_3_	Serine	105.09258	0.1051
		C_4_H_9_NO_3_	Threonine	119.11916	0.1191
		C_11_H_12_N_2_O_2_	Tryptophan	204.22518	0.02042
		C_5_H_11_NO_2_	Valine	117.14634	0.1051
	Organic acids	C_6_H_8_O_7_	Citrate	192.12352	0.2101
		C_4_H_2_O_4_Na_2_	Fumarate (disodium salt)	160.0358186	0.16
		C_4_H_6_O_5_	Malate	134.08744	0.1341
		C_3_H_4_O_3_	Pyruvate	88.06206	0.0881
		C_4_H_4_Na_2_O_4_	Succinate (disodium salt)	162.0516986	0.2701
	Base	C_4_H_5_N_3_O	Cytosine	111.102	0.1111
		C_4_H_4_N_2_O_2_	Uracil	112.08676	0.1121

**Table 2:** Specific rates for the 3 sets of conditions.

C-sources	*μ*_max⁡_ (h^−1^)	*r*_*s*_ (g substrate/gDW h)	*r*_*s*_ (C-mmole/gDW h)
Glucose	0.391	0.304	10.133
Ethanol	0.121	0.027	1.117
Glycerol	0.129	0.048	1.564

**Table 3:** List of major metabolites (significantly correlate response over time in the different C-source) by positive ESI mode.

Positive ESI pairs		PC
homoserine/threonine	Carnitine/4-aminobutanoate	1
Methylglyoxal	3-Indoleacetonitrile	
Succinate	3-Indoleacetonitrile	
Valine	3′, 5′ cyclic AMP	
Leucine	3′, 5′ cyclic AMP	
4-Aminobutyric acid/aspartate	3′, 5′ cyclic AMP	
Valine	3′, 5′ cyclic AMP	
Homoserine/threonine	3′, 5′ cyclic AMP	
Thymine/4-guanidino-butanamide	3′, 5′ cyclic AMP	
Leucine	3′, 5′ cyclic AMP	
sn-Glycerol-3-phosphate/3-dehydroshikimate	3′, 5′ cyclic AMP	
Glutamine	3′, 5′ cyclic AMP	
But-1-ene-1,2,4-tricarboxylate/7,8 diaminononanoate	3′, 5′ cyclic AMP	
Valine	3′, 5′ cyclic AMP	
Methylglyoxal	3′, 5′ cyclic AMP	
homoserine/threonine	3′, 5′ cyclic AMP	
Valine	3′, 5′ cyclic AMP	
Succinate	3′, 5′ cyclic AMP	
N-Acetyl-L-glutamate	3′, 5′ Cyclic AMP	
Leucine	Nicotinate-D-ribonucletide	
4-Aminobutyric acid/aspartate	Nicotinate-D-ribonucletide	
Glycerone/lactate	Nicotinate-D-ribonucletide	
sn-Glycerol-3-phosphate/3-dehydroshikimate	Nicotinate-D-ribonucletide	
But-1-ene-1,2,4-tricarboxylate/7,8 diaminononanoate	Nicotinate-D-ribonucletide	

Leucine	O-Acetyl-L-serine	2
Aspartate	O-Acetyl-L-serine	
Leucine	O-Acetyl-L-serine	

**Table 4:** List of major metabolites (significantly correlate response over time in the different C-source) by negative ESI mode.

Negative ESI pairs		PC
Glyoxylate/(s)-lactaldehyde	4-Hydroxybenzoate	1
Ser	Itaconate/N-acetylputrescine	
4-Aminobutanoate	Xanthine	
4-Aminobutanoate	Carnitine	
Glycerone/lactate	3-(-4-hydroxyphenyl)pyruvate/glucose/myoinositol	
(s)-Lactaldehyde/glycerol	3-(-4-hydroxyphenyl)pyruvate/glucose/myoinositol	
Glycine	3-(-4-hydroxyphenyl)pyruvate/glucose/myoinositol	
4-Hydroxybenzoate	3-(-4-hydroxyphenyl)pyruvate/glucose/myoinositol	
2-Oxoglutarate/gamma-amino-gamma-cyanobutanoate/2-dehydropantoate/glutamine/lysine	3-(-4-hydroxyphenyl)pyruvate/glucose/myoinositol	
Itaconate/N-acetylputrescine	3-(-4-hydroxyphenyl)pyruvate/glucose/myoinositol	
4-Hydroxy-L-threonine/adenine/homocysteine/2-phenylacetamide/3-hydroxyanthranilate	3-(-4-hydroxyphenyl)pyruvate/glucose/myoinositol	
3-Hydroxyanthranilate	3-(-4-hydroxyphenyl)pyruvate/glucose/myoinositol	
(s)-lactaldehyde/glycerol	cdAMP	
Glycine/sn-glycerol 3-phosphate	cdAMP	
Itaconate/N-acetylputrescine	cdAMP	
D-Glyceraldehyde 3-phosphate/glycerone phosphate/guanine/N-methyl-L-histidine/pyridoxamine phosphate/pyridoxine	cdAMP	
3-Hydroxyanthranilate	cdAMP	

Sulfide	Glycerol	2
Sulfide	Uracil	
Glycerol	Phosphocholine	
Met	Phosphocholine	

**Table 5:** List of key metabolites in positive ESI mode.

Abbreviation	Metabolites	*m*/*z*	Suggested
ACNL	3-Indoleacetonitrile	198.1135	+CH_3_CN
ASP	Aspartate	88.0879	−CHOOH
cAMP	Cyclic AMP	312.4857 330.0861	−H_2_O+H
DANNA	7,8 Diaminonanoate	189.0893	+H
DHSK	3-Dehydroshikimate	173.1333	+H
GBAD	4-Guanidinine-butanide	127.1331	−H_2_O
GL3P	sn-Glycerol-3-phosphate	173.1333	+H
GLN	Glutamine	188.0823	+CH_3_CN
HSER	Homoserine	102.0638	−H_2_O
LEU	Leucine	86.1026 and 132.1146	−N?, −CHOOH,
MTHGXL	Methylglyoxal	73.0893	+H
NAGLU	N-Acetyl-L-glutamate	190.069	+H
NAMN	Nicotinate-D-ribonucleotide	378.0777	+CH_3_CN
OAHSER	O-Acetyl-L-Serine	136.4695	+H
SUCC	Succinate	119.0985	+H
THR	Threonine	102.4362 and 120.4362	−H_2_O +H
THY	Thymine	127.1331	+H
VAL	Valine	72.0881 and 118.0985	−CHOOH +H

**Table 6:** List of key metabolites in negative ESI mode.

Abbreviation	Metabolites	*m*/*z*	Suggested
4HBZ	4-Hydroxybebzoate	119.0248	−H_2_O
4HLT	4-Hydroxy-L-threonine	134.3312	−H
AD	Adenine	134.3312	−H
AKG	2-Oxoglutarate	127.0434	−H_2_O
GABA	4-Aminobutanoate	102.4149	−H
GL	Glycerol	73.0310 and 91.0174	−H_2_O −H
GLC	Glucose	179.0353	−H
GLX	Glyoxalate	73.0310	−H
GLY	Glycine	76.0346	−H
GN	Guanine	150.3323	−H
HAN	3-Hydroxyanthranilate	152.0691	−H
HCYS	Homocysteine	134.3312	−H
LACAL	Lactaldehyde	73.0310	−H
LYS	Lysine	127.0434	−H_2_O
MET	Methionine	148.0470	−H
MVL	Mevalonate	129.0356	–H_2_O
MHIS	N-Methyl-L-histidine	150.3223	−H_2_O
PCHO	Choline Phosphate	183.0650	−H
PDLASP	Pyridoxamine phosphate	150.3223	−H_3_PO_4_
cdAMP	Cyclic dAMP	215.0070	−H_3_PO_4_
PYRDX	Pyridoxine	150.3223	−H_2_O
SER	Serine	104.1285	−H
H2SO3	Sulfide	80.9743	−H
T3P1	D-Glyceraldehyde-3-phosphate	150.3223	−H_2_O
T3P2	Glyceronephosphate	150.3223	−H_2_O
URA	Uracil	93.0162	−H_2_O
XAN	Xanthine	151.0958	−H
